# Assafœtida in Whooping Cough

**Published:** 1871-01

**Authors:** 


Assafœtida in Whooping Cough.—This has long been a familiar remedy in Whooping-Cough. It was a favorite with Dr. Chapman, especially in combination with one of the alkalies. It is best adapted to the second and third stages of the disease. It may be given with great efficacy either by the mouth or per anum.

